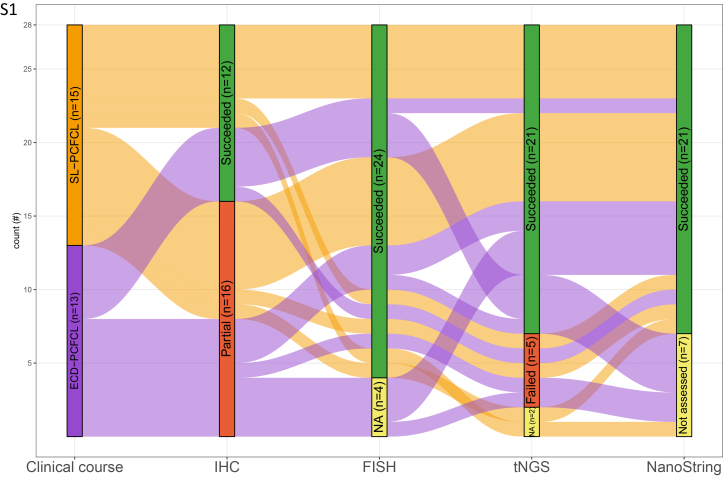# IgM Immunohistochemical Expression is a Potential Risk Factor for Extracutaneous Dissemination in Patients With Primary Cutaneous Follicle Center Lymphoma

**DOI:** 10.1097/PAS.0000000000002436

**Published:** 2025-06-13

**Authors:** Anne M.R. Schrader, Ruben A.L. de Groen, Rein Willemze, Patty M. Jansen, Koen D. Quint, Tom van Wezel, Ronald van Eijk, Dina Ruano, Cornelis P. Tensen, Arjan Diepstra, Anke van den Berg, Lianne Koens, Naomi Kakiailatu, Maarten H. Vermeer, Joost S.P. Vermaat

**Affiliations:** * Departments of Pathology; † Hematology, and; ‡ Dermatology, Leiden University Medical Center, Leiden; § Department of Pathology, University Medical Center Groningen, Groningen; ∥ Department of Pathology, Amsterdam University Medical Center, Amsterdam, The Netherlands

**Keywords:** primary cutaneous follicle center lymphoma, extracutaneous dissemination, genetic profile, IgM, MYD88

## Abstract

Primary cutaneous follicle center lymphoma (PCFCL) is a type of cutaneous B-cell lymphoma with an indolent behavior and a 5-year disease-specific survival of 95%. Given the difficulty of identifying patients at risk for developing extracutaneous dissemination (ECD), this study aimed to identify predictors in the clinical presentation, histopathology, immune phenotype, and genetic profile of PCFCL patients by comparing those who developed ECD with those whose disease remained skin limited (SL) during follow-up. After review of clinical data and histopathology, a total of 13 ECD-PCFCL patients and 15 SL-PCFCL patients with varying treatments were included from the Dutch Cutaneous Lymphomas Registry. At diagnosis, all patients presented with classic PCFCL lesions on the trunk or head-and-neck region, and histology indicated a predominance of centrocytes admixed with centroblasts. IgM expression was significantly more frequent in ECD-PCFCL (54%) than in SL-PCFCL (7%; *P*=0.006). Targeted next-generation sequencing (NGS) with a 200 B-cell lymphoma-related gene panel demonstrated known PCFCL-like mutations in both groups. In addition, ECD-PCFCL demonstrated an enrichment of mutations associated with the activated B-cell genotype, including *MYD88* (n=2), and some unique mutations, such as in ERBB4 (n=4). In conclusion, this study identified IgM expression at diagnosis as a potential biomarker for extracutaneous spread in PCFCL. In IgM-positive cases, genetic testing may be warranted. Patients with uncommon mutational profiles, such as those resembling the ABC-DLBCL genotype, may particularly benefit from closer follow-up and consideration of more aggressive treatment, including immuno-polychemotherapy. As these observations were made in a limited number of patients, our results require validation in an independent cohort.

Primary cutaneous follicle center lymphoma (PCFCL) is an extranodal B-cell non-Hodgkin lymphoma that presents with cutaneous lesions only at time of diagnoses.^1^ Patients mainly present with solitary or grouped plaques and tumors, surrounded by erythema, located in the head-and-neck region or on the trunk. Histologically, PCFCL shows dermal infiltration of centrocytic and centroblastic cells with a follicular, a diffuse, or a mixed follicular and diffuse growth pattern.^1^ The tumor cells characteristically express BCL6, are negative for MUM1, IgM, and BCL2, and show variable expression of CD10, mostly in cases with a follicular growth pattern.^2,3^ The genetic profile of PCFCL patients is heterogeneous, but predominated by loss-of-function mutations in the immunomodulatory genes *TNFRSF14* (24% to 40%) and *IRF8* (0% to 17%), in the transcription factors *TNFAIP3* (25%), *MYC* (17%), and *FOXO1* (17%), in the chromatin modifiers *CREBBP* (17% to 25%), *KMT2D* (21% to 22%), *EP300* (18%), *EZH2* (11%), *SETD2* (11%), and histone H1 genes (50%), and in the JAK-STAT signaling genes *SOCS1* (20%), *STAT6* (17%).^4–6^ *MYD88* L265P mutations and *CDKN2A* deletions, both frequently present in primary cutaneous diffuse large B-cell lymphoma, leg type (PCDLBCL-LT), appear to be absent in PCFCL.^7,8^ *BCL2* rearrangements, characteristic for systemic follicular lymphoma (FL), are rare (∼10%) in PCFCL patients.^6,9,10^ In addition, rearrangements of the *MYC* gene are even rarer and have only been reported in 2 PCFCL patients.^11–14^ Finally, gene-expression profiling of PCFCL revealed similarities to the germinal center B-cell-like subtype of diffuse large B-cell lymphoma.^15,16^

In general, PCFCL has an indolent behavior.^3^ Local radiotherapy is the preferred treatment in most PCFCL patients, resulting in a complete remission of the treated lesion in 99% of the patients.^17^ Cutaneous relapses are frequent (30%) and not associated with an adverse prognosis; however, 10% of the patients develop extracutaneous dissemination (ECD), resulting in a disease-specific survival of 95%.^3,18^ Adverse prognostic factors in PCFCL are scarce and treatment with immunochemotherapy (R-CHOP) in the first line is only recommended in case of disease localization on the leg(s).^3^

Here, we evaluated the clinical presentation, histopathology, immune phenotype, and genetic profile of the largest cohort described in the literature of PCFCL patients who did and who did not develop ECD during follow-up to identify possible risk factors for developing ECD and an adverse outcome.

## MATERIALS AND METHODS

### Patient Selection

PCFCL patients, diagnosed between 1985 and 2018, who developed ECD during follow-up were identified from the Registry of the Dutch Cutaneous Lymphoma Group and included in this study. In addition, a similar number of patients with skin-limited disease (SL-PCFCL) with at least 5 years of follow-up were included as the control group, roughly matched for sex and age at diagnosis. Initial diagnosis of all patients was made during one of the regular meetings of the Dutch Cutaneous Lymphoma Group and revised for the purpose of this study by P.M.J., R.W., and A.M.R.S., according to the 2016 World Health Organization (WHO) and 2018 WHO—European Organization for Research and Treatment of Cancer (EORTC) classification system for cutaneous lymphomas.^1^ Staging procedures -either consisting of a PET-CT scan or a combination of a CT scan and a bone marrow biopsy—were performed in all included patients and demonstrated no evidence of disease in extracutaneous sites at the time of diagnosis. The clinical data (sex, age at diagnosis, site(s) of involvement, baseline lactate dehydrogenase (LDH) levels, treatment, recurrent disease [including sites], follow-up duration, and disease status at last follow-up) were collected from the Registry of the Dutch Cutaneous Lymphoma Group and/or from the medical files. Formalin-fixed and paraffin-embedded (FFPE) tissue blocks from representative skin lesions were obtained from the Dermatology Biobank. In ECD-PCFCL, FFPE biopsies from ECD sites were also included if available. Patients were excluded in case of insufficient or no available FFPE skin biopsies. The study was performed in accordance with the Dutch Code for Proper Secondary Use of Left-Over Tissue and provided with a waiver of consent by the Medical Ethics Committee of the Leiden University Medical Center, Leiden, The Netherlands (B19.018).

### Immunohistochemistry

Immunohistochemistry was routinely, or in case missing for the purpose of this study, performed for CD10 (clone 56c6, 1:20 dilution; Dako), BCL6 (clone PG-B6p, 1:100 dilution; Invitrogen), MUM1 (clone MUM1p, 1:100 dilution; Dako), BCL2 (clone 124, 1:80 dilution; Dako), MYC (clone Y69, 1:100 dilution; ABCAM), IgM (polyclonal, 1:500 dilution; Dako) Ki67 (clone MIB-1, ready-to-use), and CD21 (clone 1F8, ready-to-use) or CD35 (clone BERMACDRC, dilution 1:10) with the Dako Autostainer Link 48 (Dako), according to standard procedures. The threshold for markers to be scored positive was any expression of CD21 or CD35 for presence of follicular dendritic cell (FDC)-networks and expression in >30% of the tumor cells for CD10, BCL6, Ki67, and MUM1, >40% of the tumor cells for MYC, and >50% of the tumor cells for BCL2 and IgM. Scoring was performed by A.M.R.S., P.M.J., and R.W., blinded for the clinical data, until consensus was reached.

### Fluorescence in situ Hybridization

Fluorescence in situ hybridization (FISH) was performed for *MYC*, and, in case of a *MYC* rearrangement, also for *BCL2* and *BCL6*, using Vysis Dual Color Break Apart Rearrangement Probes (Abbott) and the Dako Histology FISH Accessory Kit (Dako), according to the manufacturer’s recommendations. In addition, *BCL2* rearrangement status was assessed in the case of immunohistochemical expression of BCL2. A case was considered positive in case of a split of the signals in ≥10% of a minimum of 100 tumor cells scored. The rearrangement *s*tatus of 2 patients with progressive disease and 10 patients with indolent disease was previously described.^13^

### Nucleic Acid Isolation

Total nucleic acids (DNA/RNA) were automatically isolated from 10 μm microdissected FFPE tissue sections with the Tissue Preparation System (TPS) robot (Siemens Health care Diagnostics), as described previously.^19^ DNA concentrations were quantified with the Qubit dsDNA HS Assay Kit and RNA was quantified with the Qubit RNA BR Assay Kit with the Qubit Fluorometer (Invitrogen), according to standard procedures.

### NanoString Analysis–Lymph2Cx Probe Set

Gene-expression profiling (GEP), using ∼150 ng (range: 100 to 200 ng) of RNA per sample, was performed on the NanoString platform with the Lymph2Cx panel. This panel evaluates the expression of 7 activated B-cell (ABC)-related genes, 8 germinal center B-cell (GCB)-related genes, and 5 housekeeping genes for normalization. Raw counts obtained by NanoString gene-expression analysis were uploaded at the Lymphoma/Leukemia Molecular Profiling Project (LLMPP) website for cell-of-origin categorization (https://llmpp.nih.gov/LYMPHCX/).^20^ The outcome of GEP analysis, resulting in a prediction score between 0 and 1, was previously described in 15 PCFCL patients.^21^

### Targeted Next-Generation Sequencing

Cases were sequenced with the “BLYMF200” panel, in-house designed and validated targeted next-generation sequencing (tNGS) Ion-Torrent-based custom AmpliSeq panels (ThermoFisher Scientific). The BLYMF200 panel consists of 6566 amplicons divided over 2 primer pools that cover 200 lymphoma-relevant genes. This panel is an expanded version of the LYMFv1 panel that consists of 52 genes and is implemented in the routine diagnostics of the pathology department of LUMC and has been described previously.^22^ The BLYMF200 panel was compiled from an extensive review of the available literature during the start of the year 2018. Mutational frequencies and clinical relevance of non-Hodgkin B-cell lymphomas were investigated, in particular diffuse large B-cell lymphoma. The BLYMF200 panel covers the complete proposed consensus tNGS panel for all mature lymphoid malignancies.^23^

The libraries were prepared manually according to manufacturers’ procedures and sequenced on an Ion Torrent S5-system (ThermoFisher Scientific). Sequencing reads were aligned to the human reference genome (GRCh37/hg19) using TMAP 5.07 software, with default parameters (https://github.com/iontorrent/TS).^24^ Samples were excluded if the average read count was below 100 reads, or a higher transition to transversion rate than 5 within variants ≥10% allele frequency. Variants were identified with the Torrent Variant Caller and loaded into the Geneticist Assistant NGS interpretive Workbench (SoftGenetics) for variant annotation. Variants were annotated into class 1 (benign), class 2 (likely benign), class 3 (unknown significance), class 4 (likely pathogenic), and class 5 (pathogenic).^25^ Classes 4 and 5 variants were designated as pathogenic mutations together with class 3 variants with a high CADD-phred score (>25) and/or a pathogenic prediction from ≥2 of 4 selected prediction scores (SIFT, Polyphen, LRT, and MutationTaster). Lastly, the mutational profile was used to assign each case into one of the LymphGen clusters with the online webtool (https://llmpp.nih.gov/lymphgen/lymphgendataportal.php).^26^

### Statistical Analysis

Comparison between the SL-PCFCL and ECD-PCFCL patients was performed with the Fisher exact test for categorical variables and the Student *t* test and Kruskal-Wallis test for continuous variables. The follow-up duration was defined as the time from the date of diagnosis to the date of death from any cause or to the last known date of follow-up for patients who were still alive. Statistical analyses were performed with RStudio. A *P*-value of <0.05 was considered statistically significant.

## RESULTS

**FIGURE 1:**
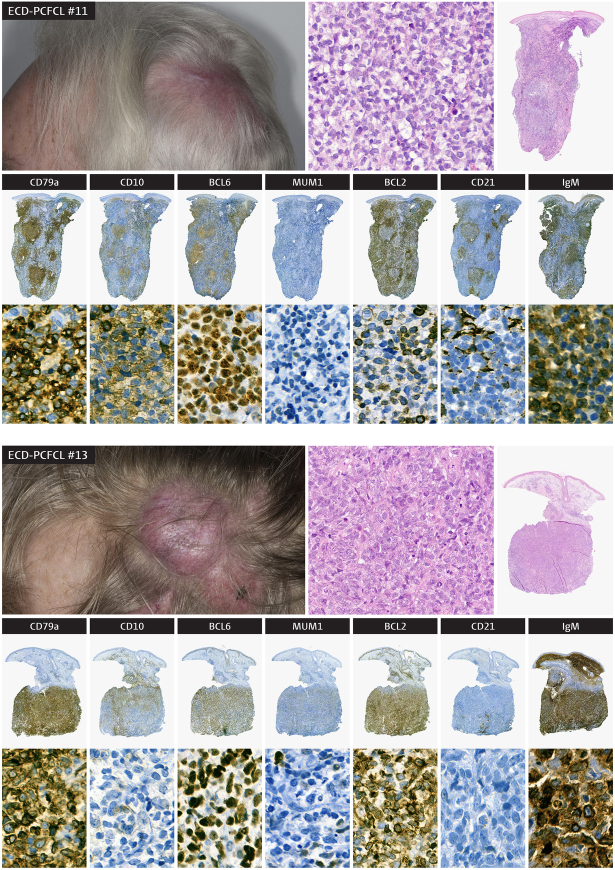
Clinical presentation and histopathology of 2 patients with primary cutaneous follicle center lymphoma (PCFCL) with extracutaneous dissemination during follow-up. Both patients presented with erythematous plaques and tumors on the scalp. Histology showed dermal infiltration of predominantly centrocytes with some centroblasts, arranges in a follicular (patient #11) and a diffuse (patient #13) pattern with expression of the tumor cells for CD79a, BCL6, and IgM, but not for MUM1, as well as presence of (remnants of) follicular dendritic cell-networks (CD21). In patient #11 with a folliculair growth pattern, also some expression of CD10 was observed, while CD10 was negative in patient #13 with a diffuse growth pattern. Both patients demonstrated expression of IgM and in patient #13 also expression of BCL2 was detected (without evidence of a BCL2 rearrangement with fluorescence in situ hybridization).

A total of 28 PCFCL patients were included: 13 patients with ECD-PCFCL and 15 patients with SL-PCFCL. The patient characteristics are presented in Table 1 and the clinical presentation and histology with immunophenotype of 2 ECD-PCFCL patients is demonstrated in Figure 1. An overview of the patient cohort and the success rate obtained with immunohistochemistry and molecular analyses is presented in Supplementary Figure 1, Supplemental Digital Content 1, http://links.lww.com/PAS/C131.

**TABLE 1:** Patient Characteristics, Immune Phenotype, and Genetic Profile of Patients With Primary Cutaneous Follicle Center Lymphoma With Extracutaneous Dissemination and Skin-Limited Disease

	All cases (n=28), n (%)	ECD-PCFCL (n=13), n (%)	SL-PCFCL (n=15), n (%)	*P* *
Clinical characteristics				
Female sex	12 (43)	7 (54)	5 (33)	0.445
Median age at diagnosis, y (range)	58 (19-71)	62 (19-71)	57 (31-66)	0.075
Disease extension				0.885
Solitary	9 (32)	5 (38)	4 (27)	
Localized	14 (50)	6 (46)	8 (53)	
Generalized	5 (18)	2 (15)	3 (20)	
Site of disease†				0.484
Upper body only	26 (93)	13 (100)	13 (87)	
Upper+lower body	2 (7)	0	2 (13)	
Lower body only	0	0	0	
Initial therapy				0.089
Intralesional steroid injection	3 (11)	3 (23)	0	
Excision	5 (18)	3 (23)	2 (13)	
Local RT	18 (64)	6 (46)	12 (80)	
Local RT + TSEB	1 (4)	0	1 (7)	
None	1 (4)	1 (8)‡	0	
** **Site of disease relapse				NA
Skin	25 (89)	12 (92)	13 (87)	
Lymph node	8 (29)	8 (62)	0	
CNS/eye	3 (11)	3 (23)	0	
Bone (marrow)	2 (7)	2 (15)	0	
Other (liver, pleura, parotic gland, pancreas)	2 (7)	2 (15)	0	
Median follow-up duration, y (range)	9.9 (2.5-17.2)	6.5 (2.5-16.1)	13.4 (5.8-17.2)	**0.006**
Status				**0.023**
Alive without disease	12 (43)	4 (31)	8 (53)	
Alive with disease	10 (36)	3 (23)	7 (47)	
Died unrelated	1 (4)	1 (8)	0	
Died of disease	5 (18)	5 (38)	0	
Histopathology				
Growth pattern				0.718
Follicular	2 (7)	1 (8)	1 (7)	
Follicular + diffuse	11 (39)	4 (31)	7 (47)	
Diffuse	15 (54)	8 (62)	7 (47)	
Immune phenotype				
CD10	20 (71)	11 (85)	9 (60)	0.221
BCL6	26 (100)§	11 (100)§	15 (100)	NA
MUM1	0║	0║	0	NA
BCL2	5 (18)	4 (31)	1 (7)	0.153
MYC	0¶	0¶	0§	NA
CD20/CD35	19 (86)#	8 (100)**	11 (79)¶	0.273
Ki67	13 (76)††	6 (75)**	7 (78)#	1.000
IgM	8 (29)	7 (54)	1 (7)	**0.006**
Genetic profile				
Cell-of-origin (lymph2Cx panel)				NA
GCB	21 (100)	7 (100)#	14 (100)¶	
ABC	0	0	0	
UI	0	0	0	
Rearrangement status				
*MYC*	1/24 (4.2)	1/9 (11.1)	0/15	**0.013**
*BCL2* ‡‡	1/8 (12.5)	0/4	1/4 (25)	0.827
*BCL6* §§	1/3 (33.3)	1/3 (33.3)	0/15	0.087
Pathogenic variants║║				
*TNFRSF14*	4 (19)¶¶	3 (30)##	1 (9)║	0.311
*CREBBP*	5 (24)¶¶	2 (20)##	3 (27)║	1.000
*EZH2*	4 (13)¶¶	1 (9)##	3 (25)║	0.587
*ERBB4*	4 (19)¶¶	4 (40)##	0║	**0.035**
*GNA13*	5 (24)¶¶	2 (20)##	3 (27)║	1.000
*ZEB2*	3 (14)¶¶	3 (30)##	0║	0.090
*MYD88*	2 (10)¶¶	2 (20)##	0║	0.214
*CD79B*	1 (5)¶¶	1 (10)##	0║	0.476
*PIM1*	1 (5)¶¶	1 (10)##	0║	0.476
Mutational profile***				0.057
GCB-associated	11 (55)	3 (30)	8 (80)	
ABC-associated	7 (35)	6 (60)	1 (10)	
Mixed	2 (10)	1 (10)	1 (10)	

* χ^2^ test for categorical data, the Mann-Whitney *U* test or Kruskal-Wallis test for continuous variables and log-rank for survival analysis. Bold values are statistically significant.

† Multiple sites per patient are possible.

‡ No initial treatment was given because of the spontaneous remission of the lesions after biopsy.

§ Data is missing in 2 cases.

║ Data is missing in 4 cases.

¶ Data is missing in 1 case.

# Data is missing in 6 cases.

** Data is missing in 5 cases.

†† Data is missing in 11 cases.

‡‡ Only tested in cases with BCL2 expression.

§§ Only tested in cases with a MYC rearrangement.

║║ Mutations with a minimum frequency of 3 cases per group, plus CD79B and PIM1.

¶¶ Data is missing in 7 cases.

## Data is missing in 3 cases.

*** Classes are defined based on the predominance of pathogenic variants associated with either the activated B-cell (ABC) or germinal center B-cell (GCB) subtype, or classified as mixed if mutations associated with both subtypes are equally present. Classes only assigned to patients with successful NGS analysis with the exception of one SL-PCFCL patient (SL08) because no differentiating mutations were detected.

ECD-PCFCL indicates extracutaneously disseminated primary cutaneous follicle center lymphoma; NA, not applicable; SL-PCFCL, skin-limited primary cutaneous follicle center lymphoma; UI, unclassified/intermediate.

### Clinical Presentation and Disease Course

The median age at diagnosis of the ECD-PCFCL patients was 62 (range: 19 to 71) years and of the SL-PCFCL patients 57 (range: 31 to 66) years. Seven (54%) ECD-PCFCL patients and 5 (33%) SL-PCFCL patients were female. In all patients, clinical presentation at the time of diagnosis consisted of characteristic lesions on the trunk or head-and-neck region. Two SL-PCFCL patients had additional lesions on the lower extremities, while this was not present in the ECD-PCFCL group. Disease presented in ECD-PCFCL and SL-PCFCL as solitary lesions in 5 (38%) and 4 (27%) patients, multiple lesions in one body region in 6 (46%) and 8 (53%) patients, and multiple lesions in more than one body region in 2 (15%) and 3 (20%) patients, respectively, without statistical significance. Also, there were no statistically significant differences in sites of involvement between the 2 subgroups (Table 1). Baseline (LDH) levels were available for 8 of 13 ECD-PCFCL patients, of whom only one had an elevated LDH at diagnosis (454 U/L). In contrast, baseline LDH levels were available for 10 of 15 SL-PCFCL patients, with elevated levels observed in 7 patients (median 360 U/L, range: 319 to 577).

In SL-PCFCL, initial treatment consisted of local radiotherapy in 12 (80%) patients, local radiotherapy combined with total head electron beam therapy in one (7%) patient, and excision in 2 (13%) patients. In ECD-PCFCL, 3 (23%) patients were initially treated with intralesional steroid injection, 3 (23%) patients with excision, and 6 (46%) patients with local radiotherapy. One of the ECD-PCFCL patients (8%) did not receive treatment because of remission of the skin lesions after biopsy. Despite no clear differences in the clinical presentation of the SL-PCFCL and ECD-PCFCL patients, initial therapy was somewhat different between the 2 groups (*P*=0.089). More specifically, the ECD-PCFCL patients were significantly less often treated with local radiotherapy than the SL-PCFCL patients in our cohort (6 vs. 13 patients; *P*=0.042). Also, initial therapy resulted in a complete remission of the skin lesions in all (100%) SL-PCFCL patients but in only 9 (69%) ECD-PCFCL cases (*P*=0.035).

The median follow-up duration was 6.5 (range: 2.5 to 16.1) years for the patients with ECD and 13.4 (range: 5.8 to 17.2) years for the SL patients. In the ECD-PCFCL group, 5 patients died from progressive disease and one patient died from cardiac disease. The remaining ECD-PCFCL patients were alive at last follow-up, including 3 patients with evidence of disease and 4 patients without evidence of disease. In the SL-PCFCL group, all patients were alive at the last follow-up, of which 7 patients had evidence of disease and 8 patients had no evidence of disease.

In ECD-PCFCL, disease extended most commonly to the lymph nodes (n=8; 62%), followed by the CNS/eye (n=3; 23%) and the bone/bone marrow (n=2; 15%). Other involved sites included the liver, pleura, parotic gland, and pancreas. In 5 ECD-PCFCL patients, the disease extended exclusively to lymph nodes, and none of these patients died of lymphoma. In contrast, the 3 patients with dissemination to the CNS, among other sites, all died of their lymphoma. The median progression-free survival (PFS) time of the ECD-PCFCL patients was 2.17 (range: 0.75 to 9.33) years. In 5 patients, the disease extended to an extracutaneous site 5 or more years after diagnosis of PCFCL, in one patient even after more than 12 years. However, a long PFS did not necessarily result in a favorable disease outcome, as 2 of these patients, with PFS times of 78 (ECD04) and 81 (ECD02) months, died of their lymphoma.

### Histology, Immune Phenotype, and Cell-of-Origin

In all cases, irrespective of the disease course, the tumor cells had a predominantly centrocytic morphology with a variable amount of admixed centroblastic cells. In one case with ECD (ECD10), a spindle-cell morphology was seen. No statistically significant difference was observed in growth pattern of ECD-PCFCL and SL-PCFCL patients, being follicular in one (8%) and one (7%) patient, combined follicular and diffuse in 4 (31%) and 7 (47%) patients, and diffuse in 8 (62%) and 7 (47%) patients, respectively.

Immunohistochemically (assessed using stated cutoffs as clarified in the Methods section), all cases were positive for BCL6, and none expressed MUM1 or MYC. The remaining immune phenotype of the patients with ECD-PCFCL and SL-PCFCL consisted of CD10 expression in 11 (85%) and 9 (60%) patients, BCL2 expression in 4 (31%) and 1 (7%) patients, and presence of (remnants of) FDC-networks in 8 (100%) and 11 (79%) patients, respectively. Interestingly, IgM was statistically significantly more often expressed in ECD-PCFCL than in SL-PCFCL patients, with 7 (54%) positive cases in ECD-PCFCL and only 1 (albeit weak) in SL-PCFCL (*P*=0.006). Lastly, the Ki67-proliferation index was more than 30% in 6 (75%) ECD-PCFCL patients and in 7 (78%) SL-PCFCL patients. Besides IgM expression, there were no statistically significant differences in immunohistochemical expression patterns between the patients with SL and ECD disease.

Cell-of-origin classification with the immunohistochemistry-based Hans algorithm and the gene-expression-based Lymph2Cx algorithm on the NanoString platform uniformly classified all cases as GCB subtypes.

### Genetic Profile

#### Gene Rearrangements

A single *MYC* rearrangement was detected in 1 of 9 (11.1%) patients with ECD-PCFCL and in none of the patients with SL-PCFCL. The *MYC*-rearranged ECD-PCFCL case (ECD10) only focally expressed MYC with immunohistochemistry with an overall expression below 40% at the time of diagnosis and, therefore, scored negative for MYC expression. In the clonally-related lymph node biopsy from this ECD-PCFCL patient, no *MYC* rearrangement was found (FISH was performed twice and both times negative). After developing ECD, this patient was treated with immune-polychemotherapy and was still in a complete remission at the last follow-up.

A *BCL2* rearrangement, only analyzed in cases with BCL2 expression or presence of a *MYC* rearrangement, was detected in 1 of 4 SL-PCFCL patients and in none of the analyzed ECD-PCFCL patients. The sole BCL2-rearranged patient was alive with evidence of disease during the last follow-up after nearly 15 years.

#### Pathogenic Variants

**FIGURE 2:**
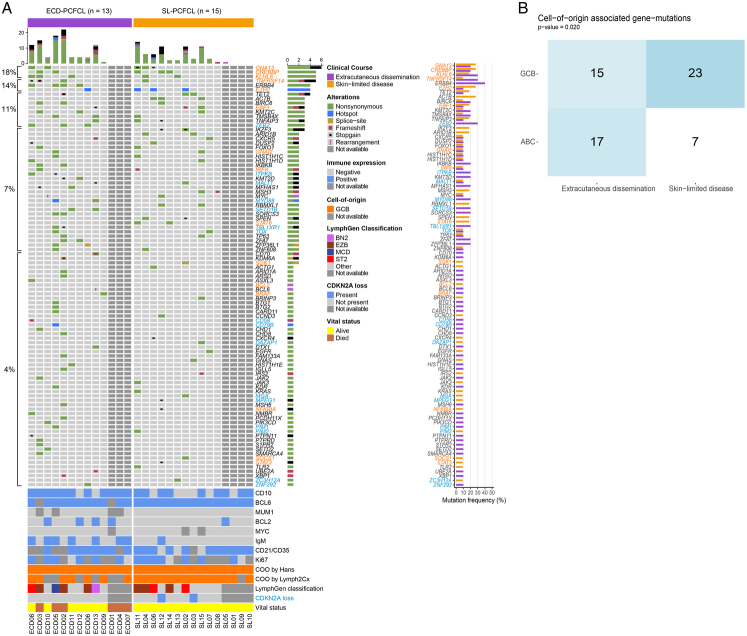
Oncoprint of the immunohistochemical and genetic profiles of patients with primary cutaneous follicle center lymphoma with extracutaneous dissemination (ECD-PCFCL; n=13) and patients with skin-limited disease (SL-PCFCL; n=15). Mutations (and their type) are represented per patient per cohort of ECD-PCFCL and SL-PCFCL. On the right side is a bar-plot for the percentage of gene-mutation in each cohort-group. Beneath the oncoprint, the metadata of each patient is presented with the immunohistochemical profile (including CD10, BCL6, MUM1, BCL2, MYC, IgM, CD21/CD35, and Ki67), NGS panel, LymphGen classification, and vital status. Assignment of genes to the ABC class (in blue) or the GCB class (in orange) was done based on the studies by Chapuy et al^27^ and Schmitz et al.^28^

Targeted NGS was successful in 10 cases with ECD-PCFCL and in 11 cases with SL-PCFCL (Fig. 2). All successfully sequenced cases harbored one or more mutations (median: 7, range: 1 to 26).

In the total cohort, mutations in *CREBBP*, *GNA13*, and *KLHL6* were most common (5/21; 24%), followed by mutations in *ERBB4*, *EZH2*, and *TNFRSF14* (4/21; 19%) and *TET2*, *ACTB*, *BIRC6*, *EBF1*, *KMT2C*, *TMSB4X*, *TFNAIP3*, and *ZEB2* (3/21; 14%). Most of these mutations were found in patients with both ECD-PCFCL and SL-PCFCL. However, *ACTB* mutations (3/11; 27%; *P*=0.214) were only present in SL-PCFCL, while *ERBB4* (4/10; 40%; *P*=0.035) and ZEB2 (3/10; 30%; *P*=0.090) mutations were exclusively detected in ECD-PCFCL, with only *ERBB4* showing a statistically significant difference between the 2 groups. Remarkably, *MYD88* mutations were detected in 2 ECD-PCFCL patients, of which one patient with the L265P hotspot variant and one patient with the non-L265P variant L202R. The patient with the *MYD88* L265P mutation (ECD5) developed CNS involvement and died of lymphoma 42 months after diagnosis, despite second-line treatment with radiotherapy and immune-polychemotherapy (R-CHOP). The patient with the non-L265P mutation (ECD13; Fig. 1), who developed lymph node involvement and was also treated with radiotherapy and immune-polychemotherapy (R-CHOP), is still alive after 45 months of follow-up. Mutations in *MYD88* L265P co-occurred with mutations in *PIM1* and *TBL1XR1*, as well as with other NF-κB genes as *CARD11* and *CD79B*, and epigenetic genes as *CREBBP*, *HIST1H1C*, and *ERBB4*. *MYD88* mutations did not co-occur with *EZH2* or *TNFRSF14* mutations, which are mostly found in B-cell lymphomas of the germinal center as opposed to the activated B-cell lymphomas, in which *MYD88* is commonly mutated. According to the LymphGen algorithm, the ECD-PCFCL patient with the *MYD88* L265P mutation was designated as belonging to the MCD cluster and the *MYD88* non-L265P as BN2, while the majority of PCFCL were either designated as EZB (29%) or ST2 (14%) clusters.

#### Paired Analysis

**FIGURE 3:**
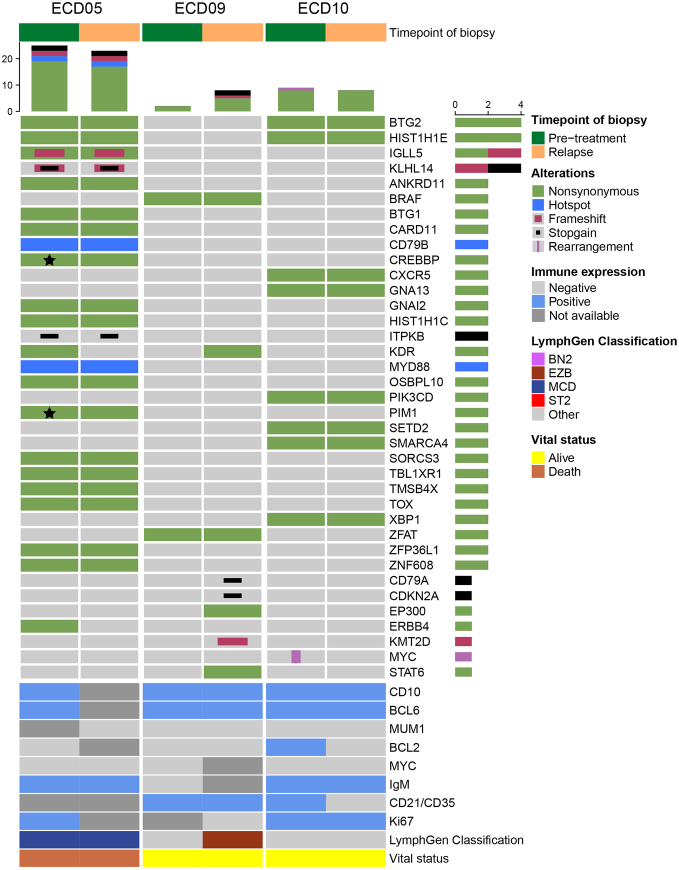
Oncoprint of the molecular profiles of paired-analysis of patients with primary cutaneous follicle center lymphoma with extracutaneous dissemination (n=3). Mutations (and their type) are represented per patient-pair. Most of the identified alterations (∼75%) were present in both biopsies. Over time, about 10% of the variants were lost and 17% were gained. The star symbol (*) indicates genes that attained more than one mutation that was lost after the patient relapsed with extracutaneous disease. Beneath the oncoprint, the metadata of each patient is presented with the immunohistochemical profile (including CD10, BCL6, MUM1, BCL2, MYC, IgM, CD21/CD35, and Ki67), LymphGen classification, and vital status.

In addition to the general molecular profile, paired analysis of skin biopsies at diagnosis and (extra)cutaneous sites at time of dissemination was performed in 3 ECD-PCFCL patients (Fig. 3). The (extra)cutaneous biopsies were obtained from involved lymph nodes (ECD09 and ECD10) and a cutaneous relapse at the time of CNS involvement (ECD5). Most of the variants (73%, n=49) identified in the diagnostic samples were also detected in the (extra)cutaneous biopsies at the time of dissemination, including mutations in NF-κB genes (eg, C*ARD11*, *CD79B*, *MYD88*, *PIM1*, and *TBL1XR1*). In contrast, some mutations, in *CREBBP*, *ERBB4*, *KDR*, and *PIM1*, and, as discussed previously, rearrangement in *MYC* were only present in the diagnostic skin lesions (10%), while mutations in genes like *CD79A*, *CDKN2A*, *EP300*, *KDR*, *KMT2D*, and *STAT6* were only detected in biopsies from (extra)cutaneous sites at time of dissemination (17%).

## DISCUSSION

In this study, we analyzed the clinical presentation, histopathology, immune phenotype, and genetic profile of 13 ECD-PCFCL patients and compared this with 15 SL-PCFCL patients with at least 5 years of follow-up to identify potential risk factors for extracutaneous dissemination in PCFCL patients.

In our cohort, all cases showed clinical and histologic features consistent with PCFCL. Also, there were no differences in disease extent, site of involvement, and the growth pattern between SL-PCFCL and ECD-PCFCL. However, IgM expression was significantly more frequent in ECD-PCFCL (54%) than in SL-PCFCL (7%; *P*=0.006). The typical immunophenotype of PCFCL is characterized by BCL6 expression and lack of BCL2, with IgM expression being rare (8% to 9%).^2^ Previous studies have suggested IgM as a marker to differentiate PCFCL from PCDLBCL-LT, where it is more commonly expressed (44% to 100%).^2,29^ The finding of IgM expression as a possible risk factor for ECD in PCFCL patients aligns with previous reports. Koens et al,^2^ described 5 PCFCL cases with IgM expression, including 1 patient who died of disseminated lymphoma 2.7 years after diagnosis. Similarly, Tsang et al^14^ reported a PCFCL patient with ECD who also expressed IgM. Three other studies investigating a total of 5 ECD-PCFCL patients did not include IgM expression in their analysis.^5,30,31^

In addition to IgM expression, which is associated with the activated B-cell-subtype of DLBCL,^32^ we observed an enrichment of ABC-associated gene mutations in the ECD-PCFCL group, conflicting with the uniform GCB signature as determined by the gene-expression-based Lymph2Cx algorithm. Also, some mutations were found exclusively in the ECD-PCFCL subgroup, including *ERBB4* (n=4), *ZEB2* (n=3), and *MYD88* (n=2), with *ERBB4* showing a statistically significant difference. These genes have not been previously reported as mutated in PCFCL,^5–8^ whereas in our cohort, 5 ECD-PCFCL patients demonstrated various combinations of these mutations*. ERBB4*, an epidermal growth factor, and *ZEB2*, a zinc finger E-box homeobox binding transcription factor, are well studied in other cancers but less understood in B-cell lymphomas.^33,34^ *ERBB4* mutations have been described in the majority of PCNSL cases;^35^ interestingly, 2 of 4 ECD-PCFCL patients with *ERBB4* mutations in our cohort also had CNS involvement. PCNSL is predominantly classified as the ABC subtype, and ZEB2 has also been found to be enriched in ABC-DLBCL.^36^ In addition, *MYD88* mutations, especially the hotspot L265P, are highly frequent in PCDLBCL-LT patients and systemic ABC-DLBCL.^16,29,37^ In our cohort, one patient had the L265P hotspot and another patient a non-L265P variant. In addition, *ERBB4* and *ZEB2* mutations often co-occurred with GCB-associated mutations (*EZH2* and *TNFRSF14*), which was not observed for *MYD88*. Despite the association of these gene mutations with ABC-DLBCL, none of the affected patients in our cohort met the criteria of a PCDLBCL-LT diagnosis. Whether or not these mutations are associated with extracutaneous spread in PCFCL patients remains to be confirmed in independent cohorts.

In addition to the profile at time of diagnosis, paired analysis of diagnostic skin biopsies and (extra)cutaneous sites at time of dissemination in 3 ECD-PCFCL patients revealed a mostly stable genetic profile (73% of variants unchanged), with some variants lost or gained over time but without a clear trend. This relative genetic stability suggests that the potential for extracutaneous dissemination and possible adverse outcomes in PCFCL may largely depend on (genetic) aberrations present at diagnosis, aligning with our findings on IgM expression. In addition, the site of dissemination appears to influence patient outcomes. In our cohort, dissemination to lymph nodes only (n=4) did not result in lymphoma-related deaths, whereas CNS involvement (n=3) was fatal in all cases. This suggests that not all PCFCL patients with ECD require aggressive treatment; involvement of lymph nodes only may suffice with local radiotherapy, while disease involvement of other sites, especially the CNS, necessitates immune-polychemotherapy.

Because ECD-PCFCL is rare, the genetic profile of these patients has been scarcely studied. Zhou et al^5^ proposed a prognostic algorithm to differentiate secondary cutaneous follicular lymphoma and PCFCL with systemic spread from PCFCL restricted to the skin. This algorithm is based on *BCL2* rearrangements, mutations in chromatin-modifying genes, and the Ki67-proliferation index. In our study, none of the 15 PCFCL patients with sufficient data (≥2 of the 3 markers available), including 7 ECD-PCFCL patients, were identified as being at risk of systemic spread. This aligns with a report by Li et al,^30^ describing 2 ECD-PCFCL patients from France who were similarly not identified as being at risk by the algorithm.

Although this study represents the largest cohort of ECD-PCFCL patients reported to date, it has several limitations. First, the sample size remains too small to draw definitive conclusions and our findings require validation in an independent cohort. Second, the cohort spans several decades, introducing a potential time period-related treatment bias: patients with ECD-PCFCL were included from 1985 onwards, whereas SL-PCFCL patients were included from 2005 onwards. ECD-PCFCL patients were statistically significantly less often treated with local radiotherapy compared with SL-PCFCL patients (*P*=0.042). As local radiotherapy is the current standard treatment for PCFCL, but was less frequently used in earlier decades, this disparity may have influenced the clinical course and disease outcomes, as reflected by the lower complete response rate to initial therapy (100% in SL-PCFCL vs. 69% in ECD-PCFCL; *P*=0.035). In addition, the age of the FFPE biopsies limited molecular analysis in several cases due to insufficient or degraded material. Finally, a limitation of our study is the use of MYC break-apart probes only. This approach may fail to detect certain IGH::MYC rearrangements due to variability in translocation breakpoints, which could have been identified using IGH::MYC fusion probes.^38^ Despite these challenges, this study offers valuable new insights into the rare subgroup of PCFCL cases with ECD.

In conclusion, this study identifies IgM expression at diagnosis as a potential biomarker for extracutaneous spread in PCFCL. In IgM-positive cases, genetic testing may be warranted. Patients with uncommon mutational profiles, such as those resembling the ABC-DLBCL genotype, may particularly benefit from closer follow-up and consideration of more aggressive treatment, including immuno-polychemotherapy.

## Supplementary Material

**Figure:**